# Serum LDH levels may predict poor neurological outcome after aneurysmal subarachnoid hemorrhage

**DOI:** 10.1186/s12883-023-03282-8

**Published:** 2023-06-13

**Authors:** Irene Cavalli, Claudia Stella, Timothée Stoll, Luciana Mascia, Michele Salvagno, Giacomo Coppalini, Alberto Diosdado, Marco Menozzi, Daniela Diaferia, Narcisse Ndieugnou Djangang, Fernando Oliveira, Sophie Schuind, Fabio Silvio Taccone, Elisa Gouvêa Bogossian

**Affiliations:** 1 Department of Intensive Care, Hôpital Universitaire de Bruxelles (HUB), Université Libre de Bruxelles (ULB), Route de Lennik, 8081070 Brussels, Belgium; 2 Department Medical and Surgical Science, Unit of Anesthesia and Intensive Care Medicine, Policlinico Di Sant’Orsola, Alma Mater Studiorum University of Bologna, Bologna, Italy; 3 Department of Biomedical and Neuromotor Sciences, Alma Mater Studiorum University of Bologna, Bologna, Italy; 4 Department of Neurosurgery, Hôpital Universitaire de Bruxelles (HUB), Université Libre de Bruxelles (ULB), Brussels, Belgium

**Keywords:** Lactate dehydrogenase, Subarachnoid hemorrhage, Biomarker, Outcome, Functional status

## Abstract

**Introduction:**

Serum lactate dehydrogenase (LDH) levels are often elevated in cardiovascular diseases. Their prognostic role after subarachnoid hemorrhage (SAH) remains poorly evaluated.

**Methods:**

This is a retrospective single-center study of patients with non-traumatic SAH admitted to the intensive care unit (ICU) of an University Hospital from 2007 to 2022. Exclusion criteria were pregnancy and incomplete medical records or follow-up data. Baseline information, clinical data, radiologic data, the occurrence of neurological complications as well as serum LDH levels during the first 14 days of ICU stay were collected. Unfavorable neurological outcome (UO) at 3 months was defined as a Glasgow Outcome Scale of 1–3.

**Results:**

Five hundred and forty-seven patients were included; median serum LDH values on admission and the highest LDH values during the ICU stay were 192 [160–230] IU/L and 263 [202–351] IU/L, respectively. The highest LDH value was recorded after a median of 4 [2–10] days after ICU admission. LDH levels on admission were significantly higher in patients with UO. When compared with patients with favorable outcome (FO), patients with UO had higher serum LDH values over time. In the multivariate logistic regression model, the highest LDH value over the ICU stay (OR 1.004 [95% CI 1.002 – 1.006]) was independently associated with the occurrence of UO; the area under the receiving operator (AUROC) curve for the highest LDH value over the ICU stay showed a moderate accuracy to predict UO (AUC 0.76 [95% CI 0.72–0.80]; *p* < 0.001), with an optimal threshold of > 272 IU/L (69% sensitivity and 74% specificity).

**Conclusions:**

The results in this study suggest that high serum LDH levels are associated with the occurrence of UO in SAH patients. As a readily and available biomarker, serum LDH levels should be evaluated to help with the prognostication of SAH patients.

**Supplementary Information:**

The online version contains supplementary material available at 10.1186/s12883-023-03282-8.

## Introduction

Rupture of cerebral aneurysm is a frequent cause of subarachnoid hemorrhage (SAH), although it accounts for just 5% of all stroke cases [1]. SAH remains a devastating cause of acute brain injury, as it affects mostly young people with an average age of 55 years and a good life expectancy [2, 3]. Over the past two decades, despite a decrease in overall mortality of SAH [1, 4, 5], morbidity remains still high and long-term neurological outcome is often poor among most of survivors. Indeed, complete recovery has been described in less than one-third while many of the affected patients may suffer from cognitive disfunction that may impair their quality of life and working capacity [2, 3, 6].

Currently, prediction of outcome after SAH is mainly based on the neurological clinical condition on admission, as assessed by the World Federation of Neurological Surgeons (WFNS) score, with a high grade score (i.e. IV-V) being associated with poor prognosis [7]. Nevertheless, different studies have shown that more than 20% of patients with high grade WFNS treated aggressively can recover completely, making initial resuscitation decisions very challenging for clinicians [8, 9]. With the aim of improving the prognostic accuracy of patients affected by SAH, modifications of WFNS scale [10], assessment of the WFNS scale after initial resuscitation [9] as well as different combined scores [11, 12] have been proposed. However, all these scores are mainly based on the clinical information on admission, and do not consider additional factors that may influence the prognosis during hospitalization.

Lactate dehydrogenase (LDH) is a non-specific biomarker expressed in almost all body tissue and it is an important enzyme involved in the anaerobic metabolism [13]. High serum LDH levels may be observed in the presence of tissue damage, hypoxic states and in several well-defined diseases and, in critically ill patients, may also represent a poor prognostic factor [13–16]. Moreover, high serum LDH values have been associated with the extend of cerebral damage in acute brain injured patients [17]. Recently, in a population of aSAH patients, high serum LDH values before microsurgical clipping were associated with poor neurological outcome at 3 months [18]; in a similar cohort, high serum LDH values on hospital admission were associated with the development of post operative pneumonia (POP) [19]; finally, in another cohort of aSAH patients, high serum LDH values were associated with early mortality [20].

The above-mentioned studies have been conducted almost exclusively in Asian populations, with different selection criteria; therefore, considering some potential variability among different populations, the aim of this study was to evaluate whether LDH might have a prognostic value in SAH patients.

## Methods

### Study population

This is a retrospective single-center cohort study including non-traumatic SAH patients admitted to the Intensive Care Unit (ICU) of Erasmus Hospital, Brussels, Belgium between January 2007 and August 2022. Inclusion criteria were: 1) age > 18 years; 2) diagnosis of ruptured aneurysm as the primary cause of SAH on computed tomography (CT) with angiographic confirmation (either computed tomography angiography or cerebral angiography). Exclusion criteria were: 1) pregnancy; 2) patients without 3 months follow up assessment reported in the medical records. This study was approved by Erasme Hospital’s Ethics Committee (P2019/649) that waived the need for informed consent. All methods were carried out in accordance with relevant guidelines and regulations in the declaration of Helsinki.

### Data collection

Demographic and clinical data were recorded including age, sex, history of hypertension, chronic obstructive pulmonary disease (COPD), heart disease, liver cirrhosis, chronic renal failure, cancer, immunosuppressive therapy, and previous neurological disease. Neurological status on admission was assessed by the WFNS score and the Glasgow coma scale (GCS). The severity of initial bleeding was evaluated by CT-scan and scored using the modified Fisher scale (mFisher). Severity of the disease was assessed using the Acute Physiology and Chronic Health Evaluation II (APACHE II) score and the Sequential Organ Failure Assessment (SOFA) score.

Aneurysm treatment (i.e. coiling and/or clipping) and use of neuromonitoring, including the need for intracranial pressure (ICP) monitoring, brain tissue oxygenation monitoring (PbtO_2_), continuous electroencephalogram (cEEG) and external ventricular derivation (EVD), were also recorded. We also collected the development of brain-specific complications, such as rebleeding, intracranial hypertension (ICHT), cerebral vasospasm, delayed cerebral ischemia (DCI), hydrocephalus and seizures, as previously described [21]. Management of such complications (i.e. osmotic therapy, decompressive craniectomy, oral or intra-arterial nimodipine etc.) were also recorded. Daily treatment including use of sedation, vasopressor, inotropes, extracorporeal membrane oxygenation (ECMO), and continuous renal replacement therapy (CRRT) were recorded. Serum LDH values were collected over 14 consecutive days from ICU admission, whenever available.

Neurological status at hospital discharge and the Glasgow outcome scale (GOS) at 3 months (GOS: 1 = dead, 2 = persistent vegetative state, 3 = severe disability, 4 = moderate disability, and 5 = good recovery) [22] were collected for each patient in the follow-up visit or estimated from medical reports; GOS score was dichotomized into unfavorable (UO; GOS 1–3) and favorable (FO; GOS 4 and 5) neurological outcome.

### Outcomes

The primary outcome was the prognostic value of admission or the highest LDH value over the ICU stay to predict UO. Secondary outcome was the prognostic value of LDH for in-hospital mortality.

### Statistical analysis

JASP 0.16.4 statistical software was used for data processing. Continuous data were expressed as mean (standard deviation [SD]) or median (interquartile ranges) according to data distribution. Differences between groups were performed with Student t-test or Mann–Whitney U-test for normally or non-normally distributed data respectively. Categorical data were presented as numbers (percentage [%]) and comparison between groups was performed by *X*-square test. Univariate logistic regression was run using UO as the dependent variable and factors associated with poor outcome [23, 24] as independent variables. Multivariate logistic regression was then performed with variables considered significant in the univariate analysis. Results of both univariate and multivariate logistic regression analysis were expressed as odds ratio (OR, with 95% confidence interval [CI]), and significance was taken at *p* < 0.05. A correlation analysis was also performed to exclude in the multivariate model pairs of variables that were closely related. To compare differences in the variations of LDH values over time in different subgroups, a linear mixed model was performed. The ability of LDH levels to predict UO was assessed using the receiver operating characteristic (ROC) curve and the area under the curve (AUROC) was calculated. Youden’s index was computed to assess the optimal cut-off of the LDH values for sensitivity and specificity to predict UO and in-hospital mortality. A *p* value below 0.05 was considered as significant.

## Results

### Study population

A total of 568 patients with aneurysmal SAH were identified over the study period; of those, 21 patients were excluded due to lost at 3 months follow-up, resulting in a total of 547 patients included in the final analysis. Main characteristics of the study population are shown in Table 1. Patients were predominantly female and had a mean age of 54.0 (± 13) years. The median GCS on admission was 14 [5–15], 246 (45%) patients had a WFNS 4–5 (poor grade), and 495 (90.5%) of the patients presented with a mFisher scale 3–4. The most common comorbidity was arterial hypertension. Intracranial hypertension and cerebral vasospasm were the most common neurological complication; DCI occurred in 134 (24.5%) patients. 172 (31.4%) patients died during hospital stay and 248 (45.3%) had UO.

**Table 1:** Characteristics of the studied population at admission. Data are presented as counts (%), mean (± SD) or medial (IQRs)

	**All patients** **(*n* = 547)**	**Unfavorable** **(*n* = 248)** **(GOS 1–3)**	**Favorable** **(*n* = 299)** **(GOS 4–5)**	***P* value**
Age (years), mean (± SD)	54.044 (± 12.9)	57.5 (± 12.9)	51.2 (± 12.1)	< .001
Female gender, n (%)	339 (62)	159 (60.2)	180 (64)	0.348
APACHE II, median (IQR)	12 (7; 18)	18 (13; 21)	8 (5; 11)	< .001
SOFA score, median (IQR)	4 (1; 8)	7.5 (5; 10)	2 (1; 4)	< .001
GCS, median (IQR)	14 (5; 15)	5 (3; 13)	15 (13; 15)	< .001
ICU length of stay, median (IQR)	7 (2; 15)	9 (2; 18)	6 (2; 13.5)	0.140
Hospital length of stay, mode (IQR)	18 (9; 28)	11 (2; 29.5)	20 (15; 28)	< .001
MV, median (IQR)	4 (2; 11)	4 (1; 12)	0 (0; 1)	< .001
mFisher 3–4, n (%)	495 (90.5)	239 (97.6)	256 (87.4)	< .001
WFNS 4–5, n (%)	246 (45)	182 (73.4)	64 (21.4)	< .001
Comorbidities, n (%)				
Hypertension	233 (42,6)	98 (40)	135 (45)	0.198
DM	47 (8.6)	32 (13)	15 (5)	< .001
Heart disease	63 (11.5)	37 (15)	26 (8.7)	0.022
Previous ND	38 (6.9)	20 (8.1)	18 (6)	0.342
CRF	10 (1.8)	4 (1.6)	6 (2)	0.737
Asthma/COPD	46 (8.4)	22 (8.9)	24 (8)	0.712
Immunosuppression	20 (3.7)	10 (4)	10 (3.3)	0.663
Cancer	26 (4.8)	14 (5.6)	12 (4)	0.366
Cirrhosis	6 (1.1)	4 (1.6)	2 (0.7)	0.289
Alcohol, n (%)	105 (19.2)	35 (14.1)	70 (23.4)	0.006
Smoking, n (%)	158 (28.9)	49 (19.8)	109 (36.5)	< .001
Drug abuse, n (%)	14 (2.6)	2 (0.8)	12 (4)	0.018
Treatment, n (%)				
Endovascular	405 (74)	153 (61.9)	252 (84.2)	< .001
Surgical	84 (15,4)	51 (20.6)	33 (11)	0.002
ICU management, n (%)				
Sedation	235 (43)	175 (70.6)	60 (20)	< .001
Opioids	297 (54.2)	160 (64.5)	137 (45.8)	< .001
Curare	85 (15.5)	72 (29)	13 (4.3)	< .001
Nimodipine	479 (87.6)	190 (76.6)	289 (96.7)	< .001
Vasopressor	299 (54.7)	213 (85.9)	86 (28.8)	< .001
Inotropes	88 (16.1)	70 (28.2)	18 (6)	< .001
Inhalation anesthetic	15 (2.7)	9 (3.6)	6 (2)	0.247
Epilepsy prophylaxis	372 (68)	183 (74)	189 (63)	0.007
Prophylactic nimodipine	427 (76.5)	184 (74)	240 (80.3)	0.090
Osmotic therapy	155 (27.8)	134 (54)	15 (5)	< .001
MV	315 (57.6)	228 (91.9)	87 (29)	< .001
RRT	2 (0.4)	2 (0.8)	0 (0)	0.119
ECMO	3 (0.5)	2 (0.8)	1 (0.3)	0.457
Hypotermia	51 (9.1)	47 (19)	2 (0.7)	< .001
Monitoring, n (%)				
EVD	281 (51.4)	181 (73)	100 (33.4)	< .001
ICP	278 (50.8)	185 (74.6)	93 (31.1)	< .001
LICOX	77 (14)	63 (25.4)	14 (4.7)	< .001
cEEG	307 (56.1)	161 (64.9)	146 (48.8)	< .001
Complictations, n (%)				
Epilepsy	128 (23.4)	76 (30.6)	52 (17.4)	< .001
Rebleeding	37 (6.8)	30 (12)	7 (2.3)	< .001
Hydrocephalus	186 (34)	115 (46.3)	71 (23.7)	< .001
Vasospasm	215 (39.3)	101 (40.7)	114 (38.1)	0.536
DCI	134 (24.5)	98 (39.5)	36 (12)	< .001
ICHT	214 (39.1)	172 (70)	42 (14)	< .001
Dec. craniectomy	28 (5.1)	24 (9.7)	4 (1.3)	< .001
Barbituric	71 (13)	69 (27.9)	2 (0.6)	< .001
Hyperventilation	161 (29.4)	142 (57.5)	19 (6.4)	< .001
Cisternal thrombolysis	9 (1.6)	9 (3.6)	0 (0)	< .001
IA nimodipine	93 (17)	62 (25)	31 (10.4)	< .001
Angioplasty	45 (8.2)	23 (9.3)	22 (7.4)	0.409
Induced hypertension	158 (29)	106 (42.9)	52 (17.4)	< .001

*APACHE* Acute Physiology and Chronic Health Evaluation, *SOFA* Sequential Organ Failure Assessment, *GCS* Glasgow coma scale, *ICU* Intensive care unit, *MV* Mechanical ventilation, *WFNS* World federation of neurosurgical societies, *DM* Diabetes mellitus, *ND* Neurological disease, *CRF* Chronic renal failure, *COPD* Chronic obstructive pulmonary disease, *RRT* Renal replacement therapy, *ECMO* Extracorporeal membrane oxygenation, *EVD* External ventricular drain, *ICP* Intracranial pressure, *cEEG* Continuous electroencephalogram, *DCI* Delayed cerebral ischemia, *ICHT* Intracranial hypertension, *IA* Intra-arterial

### LDH values and neurological outcome

**Fig. 1:**
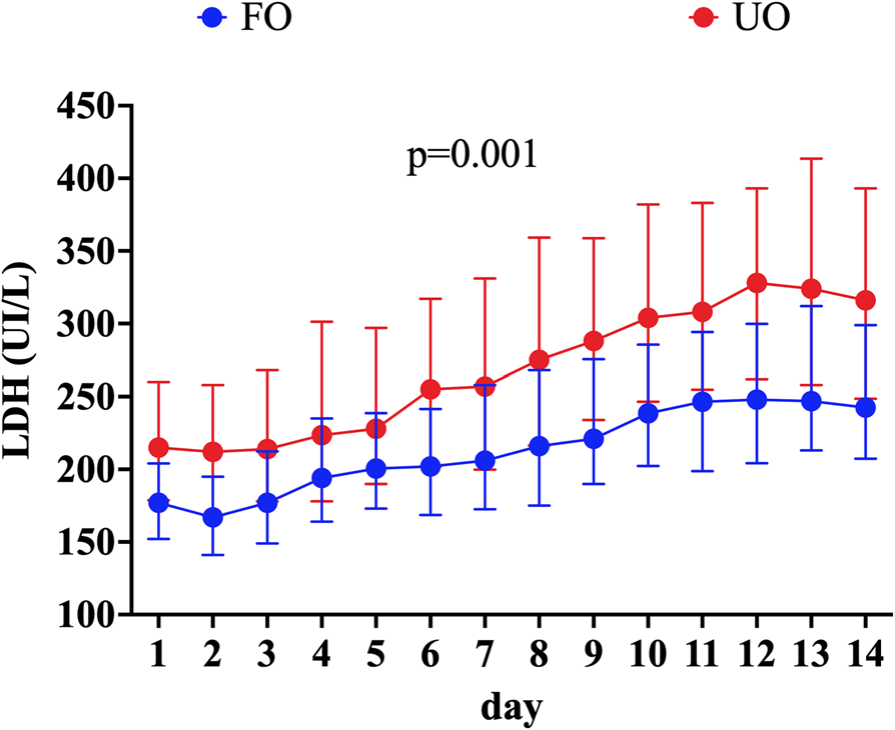
Evolution of median daily lactate dehydrogenase (LDH) levels over time in the first 14 days of hospitalization according to neurological outcome at 3 months. Unfavorable neurological outcome was defined as Glasgow outcome scale (GOS) of 1–3 and favorable neurological outcome was defined as a GOS of 4–5. *P*- value was calculated using a linear mixed model approach

Median serum LDH values on admission and the highest LDH values during the ICU stay were 192 [160–230] IU/L and 263 [202–351] IU/L, respectively. Serum LDH values on admission were significantly higher in patients with UO when compared to others (215 [179.8–260] vs. 176 [152–202] IU/L; *p* < 0.001), as well as the highest LDH value (323 [257–429] vs. 226 [159–279] IU/L; *p* < 0.001). Figure 1 and Supplemental Table 1 summarize LDH values over time, according to the neurological outcome; median serum LDH were significantly higher over time in patients with UO when compared to others.

**Fig. 2:**
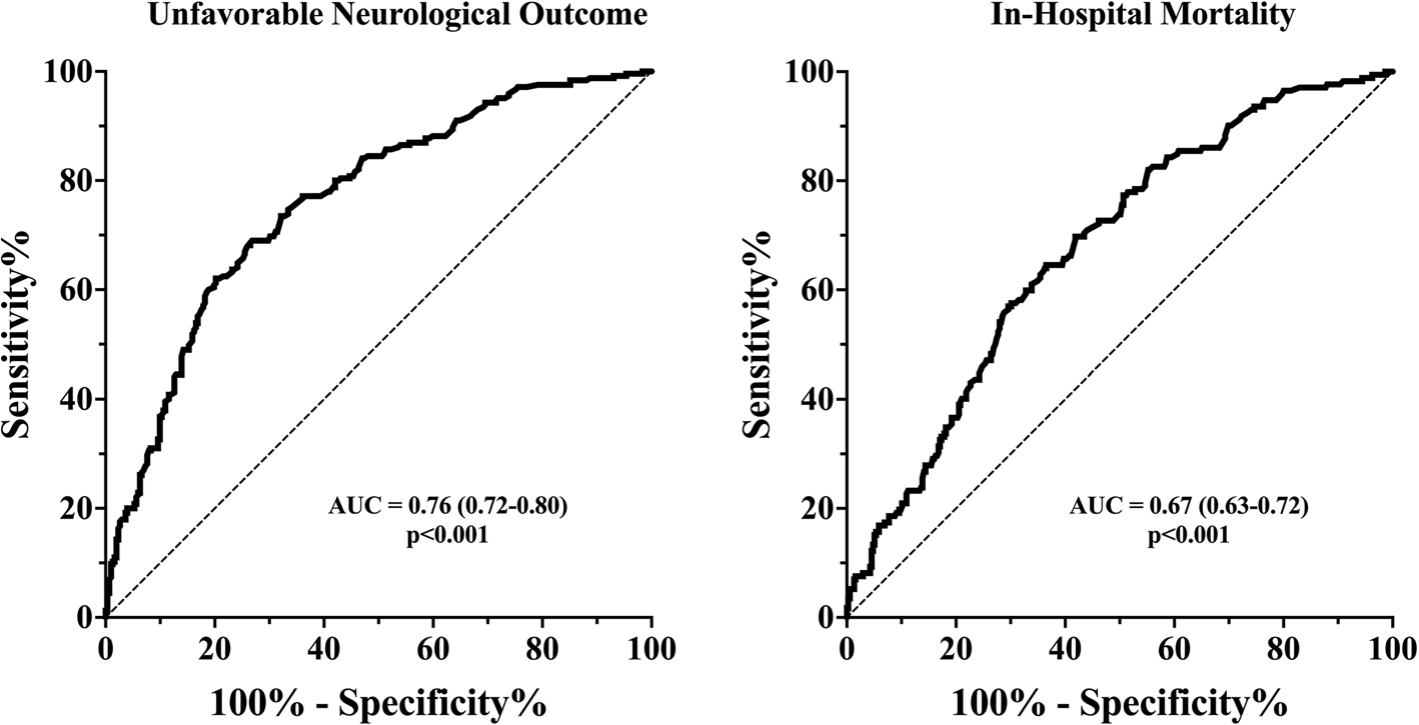
Receiver operator curve (ROC) of Lactate dehydrogenase (LDH) levels to predict unfavorable neurological outcome at 3 months and in hospital mortality. AUC: area under the curve

Patients with UO were older, had lower GCS score on admission and had more frequently an initial poor WFNS grade and higher mFisher scale than others. Also, patients with UO had higher APACHE II and SOFA score on admission and developed more frequently cerebral complications, including DCI, rebleeding, intracranial hypertension, epilepsy, and hydrocephalus than others (Table 1). In the multivariate logistic regression model, the highest LDH value over the ICU stay (OR 1.004 [95% CI 1.002 – 1.006]) was independently associated with the occurrence of UO, together with older age, WFNS score, the occurrence of DCI, ICHT and rebleeding (Table 2). The AUROC curve (Fig. 2) for the highest LDH value over the ICU stay showed a moderate accuracy to predict UO (AUC 0.76 [95% CI 0.72–0.80]; *p* < 0.001). The Youden’s index identified the threshold of the highest LDH value > 272 IU/L for the best combination of sensitivity (69%) and specificity (74%) to predict UO.

**Table 2:** Logistic regression of factors associated with unfavorable outcome (GOS 1–3) at 3 months

Variables	Univariate analysis OR [CI 95%]	Multivariate analysis OR [CI 95%]
Highest LDH	1.007 [1.005 – 1.009]	1.004 [1.002 – 1.006]
Age	1.041 [1.027 – 1.056]	1.069 [1.046 – 1.092]
WFNS	10.125 [6.824 – 15.024]	5.976 [3.483 – 10.265]
Fisher	5.757 [2.387 – 13.886]	3.340 [0.979 – 11.390]
DCI	4.773 [3.100 – 7.348]	4.373 [2.386 – 8.015]
ICHT	14.033 [9.182 – 21.448]	9.561 [5.412 – 16.892]
Hydrocephalus	2.780 [1.928 – 4.000]	0.606 [0.347 – 1.059]
Rebleeding	5.880 [2.535 – 13.638]	9.703 [2.860 – 32.920]
Epilepsy	2.099 [1.403 – 3.140]	1.175 [0.652 – 2.118]

*LDH* Lactate dehydrogenase, WFNS World Federation of Neurological Surgeons, *DCI* Delayed cerebral ischemia, *ICHT* Intracranial Hypertension

### LDH values and in-hospital mortality

Non-survivors at hospital discharge (*n* = 172) had higher serum LDH values on admission when compared with survivors (*n* = 375) (221 [187–270] vs. 180 [154–211] IU/L; *p* < 0.001), as well as the highest LDH value (310 [246–404] vs. 247 [190–320] IU/L; *p* < 0.001). Supplemental Table 2 summarize the main characteristics of the studied population, according to hospital survival; Supplemental Fig. 1 and Supplemental Table 3 summarize LDH values over time, according to hospital survival; median serum LDH were significantly higher over time in non-survivors, when compared to survivors.

In the multivariate logistic regression model, the highest LDH value over the ICU stay (OR 1.001 [95% CI 1.000 – 1.002]) was independently associated with in-hospital mortality, together with older age, WFNS score, the occurrence of DCI, rebleeding and hydrocephalus (Suppl Table 4). The AUROC curve (Fig. 2) for the highest LDH value over the ICU stay showed poor accuracy to predict in-hospital mortality (AUC 0.67 [95% CI 0.63–0.72]; *p* < 0.001).

## Discussion

In this retrospective study on a population of SAH patients, higher LDH values over the ICU stay and on admission were significantly higher in patients with poor outcome, when compared to others. Also, high LDH values over the ICU stay were independently associated with UO. The highest LDH showed a moderate accuracy to predict unfavorable outcome.

LDH is cytoplasmatic enzyme involved in the anaerobic metabolism pathway and it is present in almost all body tissue in different isomers. As such, serum LDH levels may increase in case of high tissue turnover [13]. For this reason, it is a useful and widely used biomarker in some malignancies, such as breast and lung cancer, as it could predict survival [25, 26] and response to specific therapy [27, 28]. Serum LDH levels may also increase in case of some infectious diseases, such as sepsis [29] or pneumonia [30, 31], as well as in many other pathological conditions such as liver disease, hemolytic anemia, myocardial infarction, trauma, and infections such as encephalitis, meningitis, encephalitis, and HIV [13]. Nevertheless, in SAH patients the role of serum LDH level has not been widely described and few data are available. Rupture of cerebral aneurysm results in primary brain damage due to the leakage of blood in the subarachnoid space which increases ICP and decreases cerebral blood flow [32]. This phenomenon triggers inflammatory brain reactions, pro-apoptotic and necrotic pathways that contribute to the disruption of the blood–brain barrier (BBB) and secondary brain injury [32–34]. Cell death by apoptosis or necrosis begins early after SAH and causes a release of cytoplasmic contents in the cerebrospinal fluids (CSF) [34, 35], including LDH.

The predictive value of serum LDH values is not a new finding. In 1978, Rao et al. [17] described that serum LDH levels were directly proportional to the clinical (level of consciousness) and radiological severity of brain injury, possibly representing the extent of brain damage in head injury patients. More recently, Zan et al. [20] in a retrospective study, have shown that LDH level on admission is an independent predictor of all-cause mortality in patients with SAH. In their analysis, the authors showed that for each 1-point increase in LDH, the chance of 90-days of mortality increase of 1.98 (95% CI 1.30- 3.20). Our study found an association between the highest LDH in the first 14 days of hospitalization and in hospital mortality. Another study including 647 aSAH patients reported that development of post operative pneumonia occurred more frequently in patients with serum LDH level greater than 250 U/L than in others [19]. Interestingly, in patients who underwent microsurgical clipping, pre operative LDH levels were associated with poor neurological outcome at 3 months [18]; the AUROC curve of serum LDH level on admission to predict unfavorable outcome was 0.70 (95% CI = 0.65–0.75), and the optimal cutoff value for serum LDH levels as a predictor of poor-outcome was 202 IU/L. Similarly, we also found an association between the highest LDH level in the first 14 days of hospitalization and poor neurological outcome at 3 months with an AUROC curve of AUC 0.76 [95% CI 0.72–0.80]; *p* < 0.001 with a higher LDH cutoff (272 U/L) compared to previous study [18]. Moreover, unlike the previous studies, we also considered the evolution of LDH values in the first 14 days of hospitalization, showing consistently higher LDH levels in patients with poor outcome/in-hospital mortality during early brain injury and delayed cerebral injury periods.

A retrospective study by Anan et al. [36] showed that LDH levels in CSF were higher in patients with DCI rather than in non-DCI patients but no differences in serum LDH levels were observed between the two groups. However, these results were obtained from a very small sample of only 19 patients of whom only 6 developed DCI. On the contrary, a prospective study on a population of cardiac arrest survivors [37] has shown that compared with patients with favorable outcome, both serum and CSF LDH levels were higher in patients with poor neurological outcome at 3 months evaluated by the cerebral performance category (CPC) scale. It can be assumed that serum LDH levels may be influenced by the global state of hypoperfusion as it occurs during cardiac arrest; however, both CSF and serum LDH levels follow the same trend in patients with unfavorable outcome. Even though we have studied a different population of brain injured patients and we did not collect LDH in CSF, these results are consistent with our findings.

As a readily and available biomarker, monitoring serum LDH values would be helpful for clinicians to identify patients at a higher risk of poor prognosis. In our study, the highest LDH value was observed after a median of 4 days which is before the period in which DCI can usually develop for example [38]. Thus, it would be interesting in future studies to investigate in a possible association between LDH values and neurological complications such as DCI, and whether the serum LDH level increases before such complications become clinically evident.

Our study has some limitations. First, we did not consider concurrent factors that may lead to an increase in LDH, such as the occurrence of pneumonia or sepsis, and we did not exclude patients with underlying diseases that may already present an increase in serum LDH levels, such as patients with oncological or severe liver failure. However, in our studied population, patients with oncological or severe liver disease were a negligible minority of 4,8% and 1,1% respectively. Regarding infectious pulmonary complications, the median values of MV in patients with UO were 4 days that may be too short to be consistent with the onset and resolution of a ventilator associated pneumonia for example [39, 40]. So, although we have not considered all possible confounding factors, we can speculate that since the increase in serum LDH levels are observed in patients with more severe neurological disease, that is mainly due to the SAH that promotes both neurological and systemic deterioration [41]. Second, this is a retrospective study with potential biases due to date derived from clinical records; third this is a single center study and this results may reflect local characteristics only, although our results are consistent with previous studies in different centers. Fourth, we did not measure CSF LDH levels which would be interesting to help investigate the effects of local neurological inflammation on outcome. Fifth we focused only on short term outcomes.

## Conclusion

Lactate dehydrogenase is an easily available non-specific biomarker associated with in hospital mortality and short term unfavorable neurological outcomes in non-traumatic subarachnoid hemorrhage studies. Future large multi-center studies adjusting for other causes of LDH increase such as infection are needed to better define the use of LDH as part of neuro prognostication.

## Supplementary Information

**Additional file 1:** **Supplemental Table S1.** Serum lactate dehydrogenase (LDH) values during the first 14 days in the studied population. Data are presented as median (IQRs). Unfavorable outcome (UO) was defined as Glasgow outcome scale (GOS) of 1-3 ate 3 months. Favorable outcome was defined as GOS of 4-5 at 3 months. **Supplemental Table S2.** Characteristics of the patient population, according to hospital mortality. **Supplemental Table S3.** Serum lactate dehydrogenase (LDH) values during the first 14 days in the studied population. Data are presented as median (IQRs). **Supplemental Table S4.** Logistic regression of factors associated with in hospital death. **Supplemental Figure 1.** Evolution of lactate dehydrogenase (LDH) levels over time according to hospital survival in the first 14 days of hospitalizations.

## Data Availability

All data generated or analysed during this study are included in this published article and its supplementary information files.
