# Supplementary material for: Serum LDH levels may predict poor neurological outcome after aneurysmal subarachnoid hemorrhage
Source: BMC Neurol. 2023 Jun 13;23:228. doi: 10.1186/s12883-023-03282-8 (PMC10262567; doi:10.1186/s12883-023-03282-8)
Supplement: Supplementary file 1 — Additional file 1: Supplemental Table S1. Serum lactate dehydrogenase (LDH) values during the first 14 days in the studied population. Data are presented as median (IQRs). Unfavorable outcome (UO) was defined as Glasgow outcome scale (GOS) of 1-3 ate 3 months. Favorable outcome was defined as GOS of 4-5 at 3 months. Supplemental Table S2. Characteristics of the patient population, according to hospital mortality. Supplemental Table S3. Serum lactate dehydrogenase (LDH) values during the first 14 days in the studied population. Data are presented as median (IQRs). Supplemental Table S4. Logistic regression of factors associated with in hospital death. Supplemental Figure 1. Evolution of lactate dehydrogenase (LDH) levels over time according to hospital survival in the first 14 days of hospitalizations. [file 12883_2023_3282_MOESM1_ESM.docx]

Supplemental Electronic Material

**Supplemental Table S1:** Serum lactate dehydrogenase (LDH) values during the first 14 days in the studied population. Data are presented as median (IQRs). Unfavorable outcome (UO) was defined as Glasgow outcome scale (GOS) of 1-3 ate 3 months. Favorable outcome was defined as GOS of 4-5 at 3 months.

| **LDH** | **All patients** | **UO** | **FO** | **P value** |
| --- | --- | --- | --- | --- |
| Day 1, median (IQR) | 192 (160; 231) | 215 (180; 260) | 176 (152; 202) | <0.001 |
| Day 2, median (IQR) | 180 (150; 225) | 212 (170; 258) | 167 (141; 195) | <0.001 |
| Day 3, median (IQR) | 191 (157; 240) | 214 (179; 268) | 177 (149; 211) | <0.001 |
| Day 4, median (IQR) | 206 (168; 262) | 224 (179; 301) | 194 (164; 235) | <0.001 |
| Day 5, median (IQR) | 211 (179; 268) | 228 (191; 292) | 200 (173; 239) | <0.001 |
| Day 6, median (IQR) | 225 (184; 278) | 255 (202; 316) | 202 (169; 241) | <0.001 |
| Day 7, median (IQR) | 229 (186; 301) | 257 (200; 331) | 206 (173; 257) | <0.001 |
| Day 8, median (IQR) | 245 (192; 307) | 274 (217; 351) | 214 (176; 268) | <0.001 |
| Day 9, median (IQR) | 256 (206; 328) | 288 (234; 358) | 221 (190; 274) | <0.001 |
| Day 10, median (IQR) | 264 (219; 341) | 302 (247; 379) | 238 (203; 285) | <0.001 |
| Day 11, median (IQR) | 272 (227; 355) | 306 (257; 380) | 246 (199; 290) | <0.001 |
| Day 12, median (IQR) | 289 (236; 375) | 328 (265; 393) | 248 (205; 291) | <0.001 |
| Day 13, median (IQR) | 290 (228; 367) | 328 (259; 413) | 247 (213; 311) | <0.001 |
| Day 14, median (IQR) | 280 (223; 366) | 316 (250; 391) | 242,5 (208; 299) | <0.001 |
| Highest LDH, median (IQR) | 261 (198; 350) | 322 (257; 429) | 226 (179; 278) | <0.001 |
| Day of highest LDH, median (IQR) | 4 (2; 10) | 6 (2; 11) | 4 (2; 9) | 0.014 |

**Supplemental Table S2:** Characteristics of the patient population, according to hospital mortality.

|  | **Survivors** | **Non-survivors** | **P value** |
| --- | --- | --- | --- |
| Age (years), mean (SD) | 53 (13) | 57 (14) | <.001 |
| Female sex, n (%) | 148 (40) | 60 (35) | 0.305 |
| APACHE, median (IQR) | 9 (6; 14) | 19 (15; 22) | <.001 |
| SOFA, median (IQR) | 2 (1; 5) | 8 (5; 10) | <.001 |
| GCS, median (IQR) | 15 (10; 15) | 4 (3; 10,3) | <.001 |
| ICU length of stay, median (IQR) | 8 (3; 17) | 5 (1; 11,5) | <.001 |
| Hospital length of stay, mode (IQR) | 23 (16; 39,5) | 5 (1; 12) | <.001 |
| MV, median (IQR) | 0 (0; 3) | 4 (1; 9) | <.001 |
| mFisher 3-4, n (%) | 328 (89) | 167 (98) | <.001 |
| WFNS 4-5, n (%) | 115 (31) | 131 (76) | <.001 |
| **Comorbidities n (%)**  Hypertension  DM  Heart disease  Previous ND  CRF  Asthma/COPD  Immunosuppression  Cancer  Cirrhosis | 171 (46)  27 (7)  39 (10)  24 (6)  7 (2)  27 (7)  12 (3)  13 (4)  3 (1) | 62 (36)  20 (12)  24 (14)  14 (8)  3 (2)  19 (11)  8 (5)  13 (8)  3 (2) | 0.041  0.084  0.218  0.447  0.928  0.127  0.394  0.035  0.321 |
| Alcohol, n (%) | 86 (23) | 19 (11) | 0.001 |
| Smoking, n (%) | 132 (35) | 26 (15) | <.001 |
| Drug abuse, n (%) | 13 (4) | 1 (1) | 0.048 |
| **Treatment, n (%)**  Endovascular  Surgical | 314 (84)  48 (13) | 91 (53)  36 (21) | <.001  0.013 |
| **ICU management, n (%)**  Sedation  Opioids  Curare  Nimodipine  Vasopressor  Inotropes  Inhalation anesthetic  Prophylactic antiepileptic  Vasospasm prophylaxis  Osmotic therapy  MV  RRT  ECMO  Hypothermia | 106 (28)  180 (48)  28 (8)  352 (94)  144 (38)  35 (9)  10 (3)  256 (68)  299 (80)  43 (12)  147 (39)  0 (0)  1 (0.3)  12 (3) | 129 (75)  117 (68)  57 (33)  127 (74)  155 (90)  53 (31)  5 (3)  116 (68)  125 (73)  106 (62)  168 (98)  2 (1)  2 (1)  37 (22) | <.001  <.001  <.001  <.001  <.001  <.001  0.873  0.920  0.066  <.001  <.001  0.036  0.188  <.001 |
| **Monitoring, n (%)**  EVD  ICP  LICOX  cEEG | 155 (41)  148 (40)  38 (10)  206 (55) | 126 (73)  130 (76)  39 (23)  101 (59) | <.001  <.001  <.001  0.407 |
| **Complications, n (%)**  Epilepsy  Rebleeding  Hydrocephalus  Vasospasm  DCI  ICHT  Decompressive craniectomy  Barbiturics  Hyperventilation  Cisternal thrombolysis  IA nimodipine  Angioplasty  Induced hypertension | 81 (22)  14 (4)  107 (29)  154 (41)  70 (19)  74 (120)  11 (3)  14 (4)  46 (12)  5 (1)  59 (16)  34 (9)  85 (23) | 47 (27)  23 (13)  79 (46)  61 (36)  64 (37)  140 (81)  17 (10)  57 (33)  115 (67)  4 (2)  34 (20)  11 (6)  73 (43) | 0.142  <.001  <.001  0.213  <.001  <.001  <.001  <.001  <.001  0.392  0.244  0.299  <.001 |

APACHE: Acute Physiology and Chronic Health Evaluation; SOFA: sequential Organ Failure Assessment; GCS: Glasgow coma scale; ICU: Intensive care unit; MV: mechanical ventilation; WFNS: world federation of neurosurgical societies; DM: diabetes mellitus; ND: neurological disease; CRF: chronic renal failure; COPD: chronic obstructive pulmonary disease; RRT: renal replacement therapy; ECMO: extracorporeal membrane oxygenation; EVD: external ventricular drain; ICP: intracranial pressure; cEEG: continuous electroencephalogram; DCI: delayed cerebral ischemia; ICHT: intracranial hypertension; IA: intra-arterial

**Supplemental Table S3:** Serum lactate dehydrogenase (LDH) values during the first 14 days in the studied population. Data are presented as median (IQRs)

| **LDH** | **Hospital Survivors** | **Hospital non-Survivors** | **P value** |
| --- | --- | --- | --- |
| Day 1, median (IQR) | 180 (154; 211) | 221 (188; 2670) | <0.001 |
| Day 2, median (IQR) | 169 (144; 200) | 223 (178; 258) | <0.001 |
| Day 3, median (IQR) | 180 (152; 217) | 227 (185; 258) | <0.001 |
| Day 4, median (IQR) | 199 (166; 246) | 231 (180; 301) | <0.001 |
| Day 5, median (IQR) | 203 (177; 260) | 235 (201; 306) | <0.001 |
| Day 6, median (IQR) | 214 (176; 260) | 259,5 (205; 324) | <0.001 |
| Day 7, median (IQR) | 221 (179; 281) | 258 (207,3; 339) | <0.001 |
| Day 8, median (IQR) | 240 (189; 293) | 276 (219; 360) | <0.001 |
| Day 9, median (IQR) | 242 (196; 307) | 290 (237; 367) | <0.001 |
| Day 10, median (IQR) | 254 (211; 325) | 306 (259; 405) | <0.001 |
| Day 11, median (IQR) | 257 (216; 338) | 306 (276; 410) | <0.001 |
| Day 12, median (IQR) | 275 (231; 354) | 325,5 (265; 399) | <0.001 |
| Day 13, median (IQR) | 278 (223; 361) | 317,5 (259; 455) | <0.001 |
| Day 14, median (IQR) | 270 (218; 350) | 310 (264;401) | <0.001 |
| Highest LDH, median (IQR) | 247 (190; 320) | 310 (246; 404) | <0.001 |
| Day of highest LDH, median (IQR) | 5 (2; 10) | 4 (1; 9) | 0.014 |

**Supplemental Table S4: Logistic regression of factors associated with in hospital death**

| **Variables** | **Univariate logistic**  **OR [CI 95%]** | **Multivariate analysis**  **OR [CI 95%]** |
| --- | --- | --- |
| Highest LDH | 1.003 [1.002 – 1.004] | 1.001 [1.000 – 1.002] |
| Age | 1.028 [1.013 – 1.043] | 1.035 [1.015 – 1.055] |
| WFNS | 7.224 [4.776 – 10.926] | 3.036 [1.793 – 5.139] |
| Fisher | 6.789 [2.069 – 22.269] | 2.988 [0.785 – 11.374] |
| DCI | 2.582 [1.724 – 3.867] | 1.800 [1.053 – 3.080] |
| ICHT | 17.736 [11.190 – 28.113] | 11.614 [6.801 – 19.833] |
| Hydrocephalus | 2.128 [1.463 – 3.094] | 0.584 [0.347 – 0.985] |
| Rebleeding | 3.980 [1.994 – 7.946] | 2.723 [1.064 – 6.970] |
| Epilepsy | 1.365 [0.900 – 2.069] | 0.821 [0.474 – 1.424] |

LDH: Lactate dehydrogenase; WFNS: World Federation of Neurological Surgeons; DCI: Delayed cerebral ischemia; ICHT: intracranial Hypertension

**Supplemental Figure 1:** Evolution of lactate dehydrogenase (LDH) levels over time according to hospital survival in the first 14 days of hospitalizations.

**
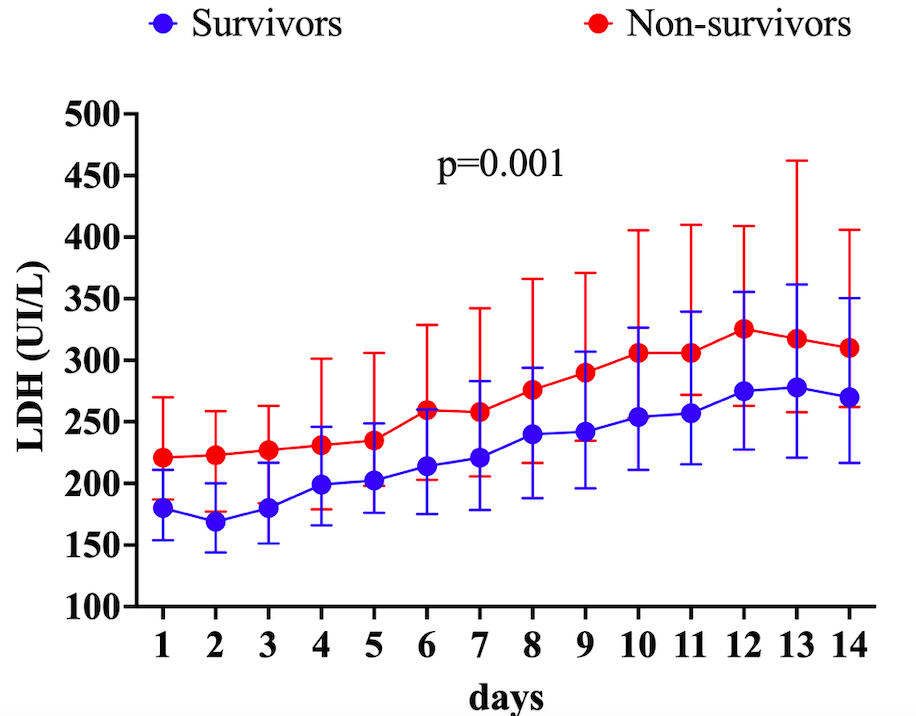
**
